# Cationic Polyamidoamine Dendrimers as Modulators of EGFR Signaling *In Vitro* and *In Vivo*

**DOI:** 10.1371/journal.pone.0132215

**Published:** 2015-07-13

**Authors:** Saghir Akhtar, Bashayer Al-Zaid, Ahmed Z. El-Hashim, Bindu Chandrasekhar, Sreeja Attur, Mariam H. M. Yousif, Ibrahim F. Benter

**Affiliations:** 1 Department of Pharmacology and Toxicology, Faculty of Medicine Kuwait University, Safat 13110, Jabriya, Kuwait; 2 Department of Pharmacology and Therapeutics, Faculty of Pharmacy, Kuwait University, Safat 13110, Jabriya, Kuwait; 3 Faculty of Medicine, Eastern Mediterranean University, Famagusta, North Cyprus; University of Central Florida, UNITED STATES

## Abstract

Cationic polyamidoamine (PAMAM) dendrimers are branch-like spherical polymers being investigated for a variety of applications in nanomedicine including nucleic acid drug delivery. Emerging evidence suggests they exhibit intrinsic biological and toxicological effects but little is known of their interactions with signal transduction pathways. We previously showed that the activated (fragmented) generation (G) 6 PAMAM dendrimer, Superfect (SF), stimulated epidermal growth factor receptor (EGFR) tyrosine kinase signaling—an important signaling cascade that regulates cell growth, survival and apoptosis- in cultured human embryonic kidney (HEK 293) cells. Here, we firstly studied the *in vitro* effects of Polyfect (PF), a non-activated (intact) G6 PAMAM dendrimer, on EGFR tyrosine kinase signaling via extracellular-regulated kinase 1/2 (ERK1/2) and p38 mitogen-activated protein kinase (MAPK) in cultured HEK 293 cells and then compared the *in vivo* effects of a single administration (10mg/kg i.p) of PF or SF on EGFR signaling in the kidneys of normal and diabetic male Wistar rats. Polyfect exhibited a dose- and time-dependent inhibition of EGFR, ERK1/2 and p38 MAPK phosphorylation in HEK-293 cells similar to AG1478, a selective EGFR inhibitor. Administration of dendrimers to non-diabetic or diabetic animals for 24h showed that PF inhibited whereas SF stimulated EGFR phosphorylation in the kidneys of both sets of animals. PF-mediated inhibition of EGFR phosphorylation as well as SF or PF-mediated apoptosis in HEK 293 cells could be significantly reversed by co-treatment with antioxidants such as tempol implying that both these effects involved an oxidative stress-dependent mechanism. These results show for the first time that SF and PF PAMAM dendrimers can differentially modulate the important EGFR signal transduction pathway *in vivo* and may represent a novel class of EGFR modulators. These findings could have important clinical implications for the use of PAMAM dendrimers in nanomedicine.

## Introduction

Cationic polyamidoamine (PAMAM) dendrimers are hyperbranched spherical polymers being investigated for a variety of applications in nanomedicine including nucleic acid drug delivery. These biomacromolecules have an ethylenediamine core and radiating branches with terminal amino (-NH2) groups whose synthesis can be precisely controlled to produce progressive generations with defined molecular architecture, terminal functional chemistry and low polydispersity [1–4]. Thus, the fifth (G5) and sixth (G6) generations of intact PAMAM dendrimers possess 128 and 256 surface amino groups, molecular weights of 28.8 and 58 kDa, with corresponding molecular diameters 5.3 and 6.7 nm respectively [5]. Following synthesis, PAMAM dendrimers can be further modified to reduce internal structural branching and increase internal volume by controlled heat-treatment in solvolytic solvents to produce so-called “fractured” or “activated” dendrimers for improved drug entrapment [6,7]. An example of a commercially available activated G6, PAMAM dendrimer is SuperFect (SF) whereas Polyfect (PF) is the intact or non-activated counterpart G6 dendrimer. As a result of their ability to bind to negatively charged nucleic acids and efficiently enter cells by endocytosis and/or via membrane pore formation [8–10], cationic PAMAM polymers have been extensively investigated for gene, oligonucleotide and siRNA delivery [3,8,10–18].

Beyond drug and nucleic acid delivery, there is emerging evidence that cationic PAMAM dendrimers, rather like some other non-viral delivery systems [19], can exhibit defined intrinsic biological (and toxicological) effects. This is highlighted by their potential investigation as, amongst others, anti-inflammatory agents for acute pancreatitis [20], anti-resistant antibiotics [21], glucose scavengers /anti-glycation agents [22–24], pore-blocking binary toxin inhibitors to counter the effects of pathogenic bacteria [25] and as nucleic acid scavengers for preventing thrombosis [26,27]. Thus, both for their safe use in the clinic and for the identification of potentially novel therapeutic applications, a detailed understanding of their biological or pharmacological actions is required. However, little is known about their biological effects in terms of their interactions with key cellular signal transduction pathways.

The epidermal growth factor receptor (EGFR) is a 175kD member of the ErbB family of receptor tyrosine kinases that, following ligand-dependent or ligand-independent activation, leads to either homo- or hetero- dimerization with related family members. This then leads to subsequent activation of several downstream effectors including Ras, Raf, extracellular-signal-regulated kinase 1/2 (ERK1/2), p38 mitogen activated protein (MAP) kinase and phosphatidylinositol 3 (PI-3) kinase/AKT (protein kinase B) pathways which are important regulators of cell growth, differentiation, survival and apoptosis [28–30]. Emerging concepts in the regulation of EGFR suggest it serves as a molecular integration site for multiple types of stimuli including peptide ligands, metal ions, ultraviolet and gamma radiation, osmotic shock, membrane depolarization, and oxidative radicals [28,31]. Acting as relay for such a wide range of stimuli highlights its importance as a key signaling pathway and gives rise to the concept that the EGFR acts as a functional keystone in “higher-order” complex systems that may underpin multifactorial global somatic actions [32]. Indeed the EGFR signaling pathway appears important in both normal and pathological states such as cancer [29] and diabetes-induced renal and cardiovascular dysfunction [33–35]. Thus, intentional or inadvertent modulation of the EGFR pathway, such as by dendrimers, could therefore have important physiological and pathological consequences.

We previously showed that the activated G6 PAMAM dendrimer, Superfect (SF), stimulated EGFR tyrosine kinase signaling in cultured human embryonic kidney (HEK-293) cells [36]. Here, we firstly studied the *in vitro* effects of naked, as supplied, non-activated (intact) G6 PAMAM dendrimer, Polyfect (PF), on EGFR tyrosine kinase signaling via extracellular-regulated kinase 1/2 (ERK1/2) and p38 mitogen-activated protein kinase (MAPK) in cultured HEK-293 cells and then compared the *in vivo* effects of a single i.p. administration of PF or SF on EGFR signaling in the kidneys of normal and diabetic male Wistar rats.

## Methods

### Drugs

Polyfect and Superfect transfection reagents were purchased from Qiagen, USA. AG1478 (*N*-(3-Chlorophenyl)-6,7-dimethoxy-4-quinazolinanine hydrochloride) and Tempol (4-hydroxy-2,2,6,6tetramethyl-piperidin-1-oxyl) were purchased from Tocris (UK). Epidermal growth factor (EGF) ligand, apocynin, catalase and all other reagents were purchased from Sigma Chemical Co (St Louis, USA).

### Cell culture and treatment with Polyfect PAMAM dendrimer

Human embryonic kidney (HEK 293) cells obtained from ECACC were a gift from Dr S Al-Sabah (Kuwait University) and cultured as described by us previously [36]. Briefly, cells were cultured in Dulbecco’s modified Eagle’s medium (DMEM) containing 4.5 mg/ml glucose, 10% heat-inactivated fetal bovine serum (FBS; Hyclone, USA), 100 mg/ml Antibiotic-Antimycotic (Invitrogen, USA), and in humidified conditions under 5% CO_2_. For the mechanistic studies, cells were plated into T25 flasks and when 60–70% confluent were treated with either 0.4, 4 or 40 μg/ml of Polyfect (used neat as supplied by the manufacturer, Qiagen Inc, USA) in serum-free media for either 0.5, 1, 4 or 24 h as stated. AG1478, a selective blocker of EGFR tyrosine kinase phosphorylation, was incubated with cells at a concentration of 10μM. Epidermal growth factor (EGF) ligand (10nM) was incubated for 30 mins in serum-free media as a positive control for stimulation of EGFR. Cells were then either used to determine cell viability or processed for Western Blotting experiments.

### Polyfect co-treatment with EGF-ligand in HEK293 cells

Overnight serum-starved HEK 293 cells, at 60–70% confluency, were incubated with EGF (10nM) for 30 mins in serum-free media for stimulation of EGFR. For co-treatment of ligand with dendrimer, PF at 0.4, 4 and 40 μg/ml was added to the serum-free media at 3.5h prior to adding EGF (10nM) for the final 30 mins. Cells were then lysed and subjected to Western Blotting.

### Treatment of HEK 293 cells with antioxidants and Polyfect

To assess the role of oxidative stress on PAMAM dendrimer-mediated effects on signaling molecules, HEK 293 cells grown to about 60–70% confluency were incubated with PF dendrimer for 4h in serum-free media in the presence or absence of antioxidants, apocynin (100 mM), catalase (2000 units/mL) or tempol (50 mM). Cells were then lysed and subjected to Western Blotting.

### Cell Viability and Apoptosis

HEK 293 cell viability was determined by trypan blue exclusion assay and cell counting performed via an automated Vi-Cell XR Cell Viablility counter (Beckman Coulter, CA, USA) that was also confirmed by direct cell counting manually using a light microscope and haemocytometer [36].

Apoptosis in HEK 293 cells was assayed using Annexin V-FITC/7-ADD kit (Beckman Coulter, IM3614) and Flow Cytometer FC 500 with CXP software (Beckman Coulter) similar to that described by us previously [37]. Briefly, cells were incubated with either PF or SF for 4h with and without Tempol. AG1478 (100 μM) was used as a positive control for inducing apoptosis. Cells were then detached by gentle trypsinisation using 0.02% EDTA-0.05% trypsin, and re-suspended in the original media to include detached cells in the suspensions. The cell suspensions were washed with ice-cold Phosphate Buffered Saline (PBS), (Sigma; P3813) and centrifuged for 5 minutes at 500 x g at 4°C. The supernatants were discarded and the cell pellets were then re-suspended in 100 μl of ice-cold 1X Binding Buffer. Approximately, 5 x10^6^ cells/ml were exposed to 10 μl of Annexin V-FITC solution and 20 μl of 7-AAD viability dye. The tubes were kept on ice and incubated in the dark for 15 minutes. Then, the cells were re-suspended in 0.4 ml ice-cold 1X binding buffer, transferred to FACS tubes (Fahrenheit, UK) and analyzed immediately using a Flow Cytometer FC 500 using CXP software (Beckman Coulter).

### Animal studies and treatments

All animal care and experimental procedures were conducted in accordance with the National Institutes of Health Guide for the Care and Use of Laboratory Animals (NIH Publication no. 85–23, Revised 1985) and were approved by Kuwait University Research Sector/Administration’s Health Sciences Research Ethics Committee following consideration of both the scientific and ethical aspects of the study design. Male Wistar rats weighing about 300g were used in this study and divided into the following groups (N = 5). Group 1: Non-diabetic (Control, C) animals, Group 2: C + PF (10mg/kg administered as a single intraperotoneal (i.p) injection) Group 3: C + SF (10mg/kg i.p); Group 4: C + AG1478 (1mg/kg i.p). Group 5: Rats bearing 4 weeks of diabetes (D) induced by a single i.p. injection of streptozotocin (55 mg/kg body weight); Group 6: D + PF (10mg/kg i.p) Group 7: D + SF (10mg/kg i.p) and Group 8: D + AG1478 (1mg/kg i.p). AG1478 and dendrimer treatments were administered as single dose for 24h prior to sacrifice based on previous studies [33,35,38,39]. Rat body weight and basal glucose levels were assessed before and after treatments just before sacrificing the animals. An automated blood glucose analyzer (Glucometer Elite XL) was used to assess blood glucose concentrations and rats with a blood glucose concentration above 250mg/dl (approx. 14 mmol/L) were declared diabetic as in previous studies [33–35].

### Histopathology of normal and diabetic rat kidneys

The kidneys isolated from non-diabetic rats or rats bearing 4-weeks of diabetes with or without treatments were fixed in formalin and mid-transverse sections taken. The fixed sections were processed for paraffin embedding, and hematoxylin-eosin–stained sections were prepared and viewed under light microscopy as described by us previously [40].

### Western Blotting studies

Western blotting for EGFR, ERK1/2 and p38 MAPK was performed essentially as described by us previously [33–36]. Briefly, either kidney tissue from *in vivo* studies or HEK 293 cell lysates were snap frozen in liquid nitrogen and stored at –80°C. Upon defrosting, protein concentrations were estimated by BioRad BCA protein assay. Aliquots containing equal amounts of protein were then subjected to SDS-polyacrylamide gel electrophoresis (SDS-PAGE) and transferred onto nitrocellulose membrane (Schleicher & Schuell, Dassel, Germany). Membranes were then incubated with either monoclonal antibodies (Cell Signaling, USA) to detect phosphorylated or total forms of EGFR (bands seen at approximately 175kDa), ERK1/2 (at 42/44 kDa) or p38 MAPK (at 38 kDa) and subsequently with appropriate secondary antibodies conjugated to horseradish peroxidase (Amersham, UK). Immunreactive bands were detected with SuperSignal chemiluminescent substrate (Pierce, UK) using Kodak autoradiography film (G.R.I., Rayne, U.K.). To ensure equal loading of proteins β-actin levels were detected using primary rabbit anti-human β-actin antibody followed by the secondary anti-rabbit IgG horse-radish peroxidase conjugated antibody (Cell Signaling, USA). Images were finally analysed and quantified by densitometry and all data normalized to β-actin levels as described previously [33–36].

### Statistical analysis

Data are presented as mean ± SEM of ‘n’ number of experiments. Mean values were compared using analysis of variance followed by post hoc test (Bonferroni). Significant difference was considered when p value was less than 0.05.

## Results

### The effect of Polyfect PAMAM Dendrimer dose on HEK 293 cell viability

**Fig 1:**
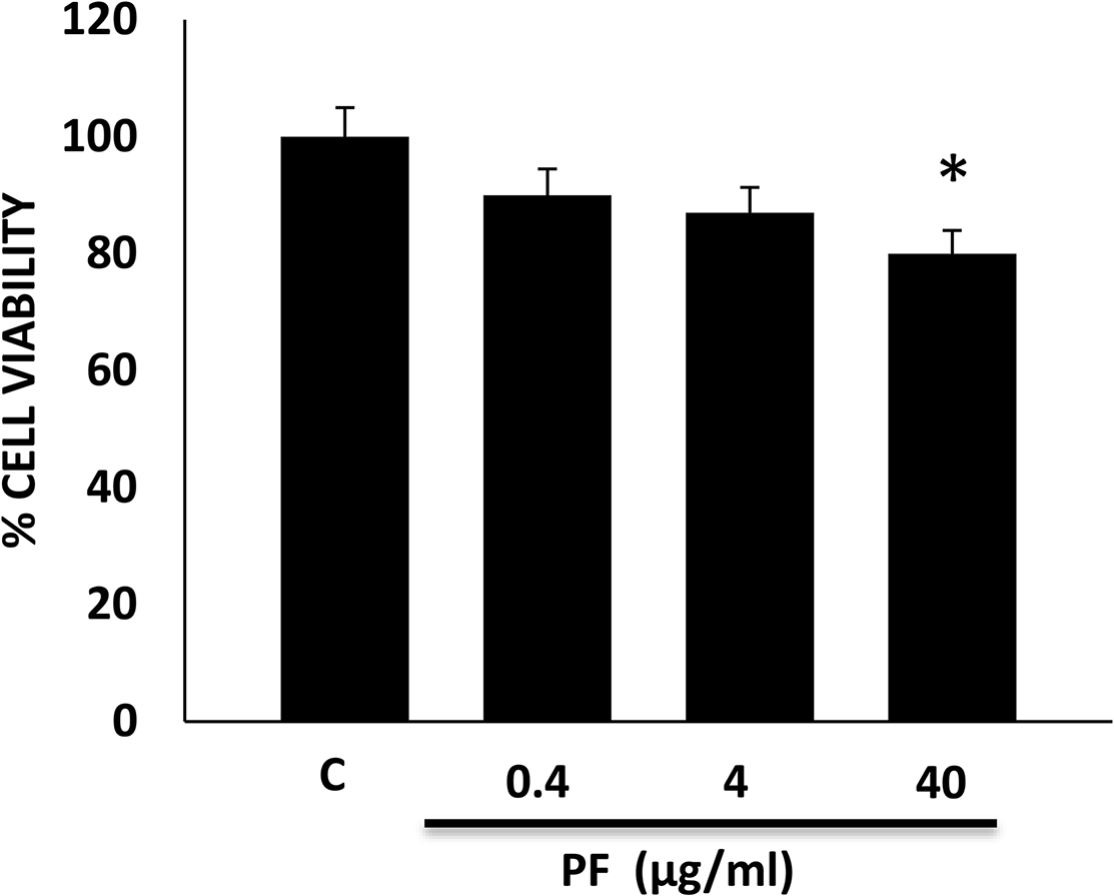
Dose-dependent effects of Polyfect (PF) PAMAM dendrimer on HEK 293 cell viability. Error bars represent mean ± SEM. * indicates statistically significant from untreated controls. N = 5.

PF dose-dependently reduced HEK 293 cell viability whereby at the highest dose of 40 μg/ml around 80% cell viability was noted (Fig 1).

### The dose-dependent effects of Polyfect on EGFR protein expression and phosphorylation

**Fig 2:**
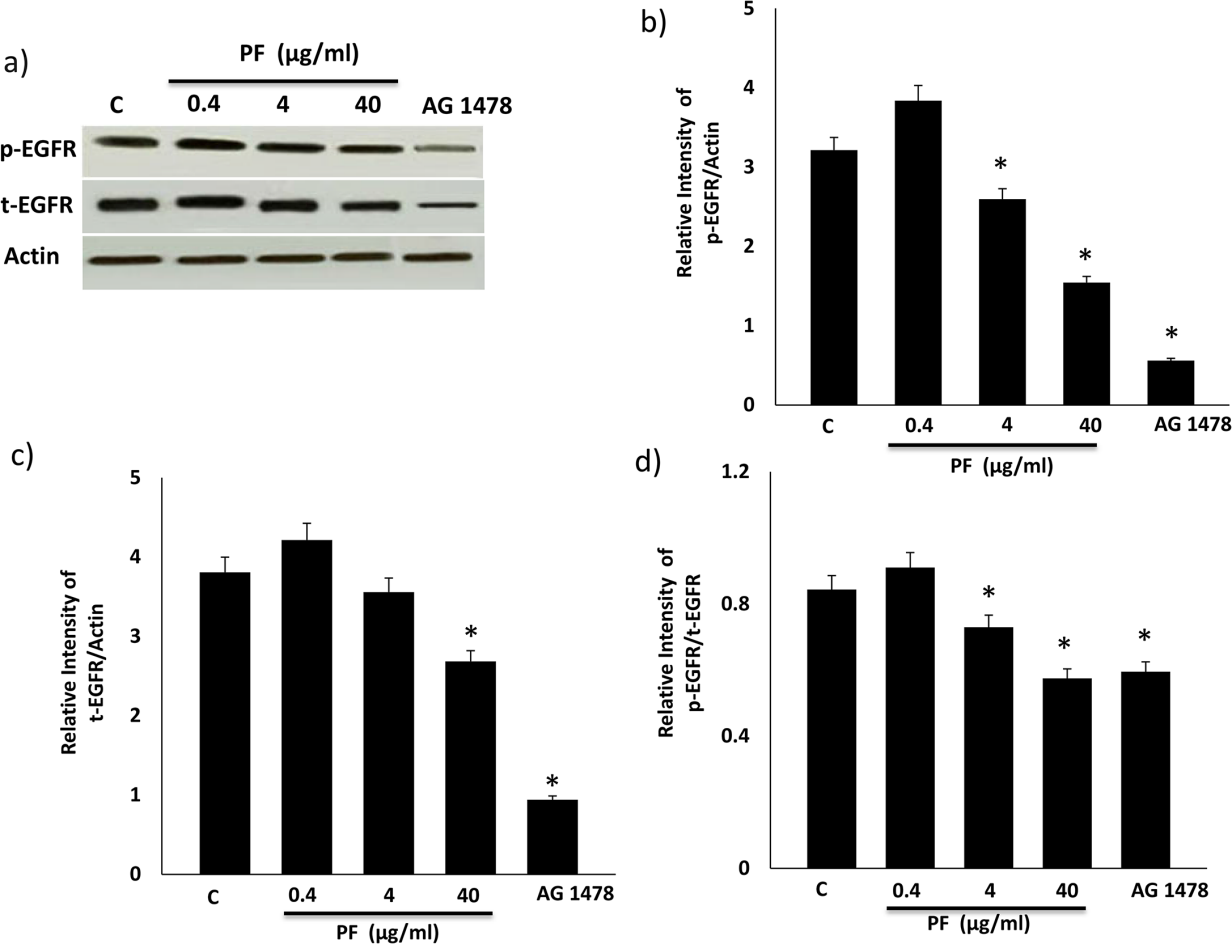
Dose-dependent effects of Polyfect (PF) PAMAM dendrimer on phosphorylated (p-) and total (t-) EGFR levels in HEK293 cells. Cells were treated with the stated doses of PF for 4h and analyzed after 24h or with AG1478 (10 μM) for 24h as per the Methods. Panel a) shows representative Western blots and panels b-d show densitometric histograms showing the relative intensity levels of pEGFR (b), total EGFR (c) bands relative to the actin loading control and the ratio of the two (panel d). Error bars represent mean ± SEM. N = 5. (Asterix) * indicates statistically significant from untreated controls.

Increasing the dose of PF led to a progressive decrease in EGFR phosphorylation levels as well as in total EGFR protein in HEK 293 cells that was statistically significant at the higher (4 or 40 μg/ml) doses (Fig 2A–2C). However, a plot of the ratio of phosphorylated to total protein showed a significant net inhibition in EGFR phosphorylation (p<0.05) which at the highest dose appeared similar to that observed with AG1478, a known selective inhibitor of EGFR phosphorylation (Fig 2D).

### Polyfect PAMAM dendrimer inhibits EGF ligand-induced stimulation of EGFR phosphorylation

**Fig 3:**
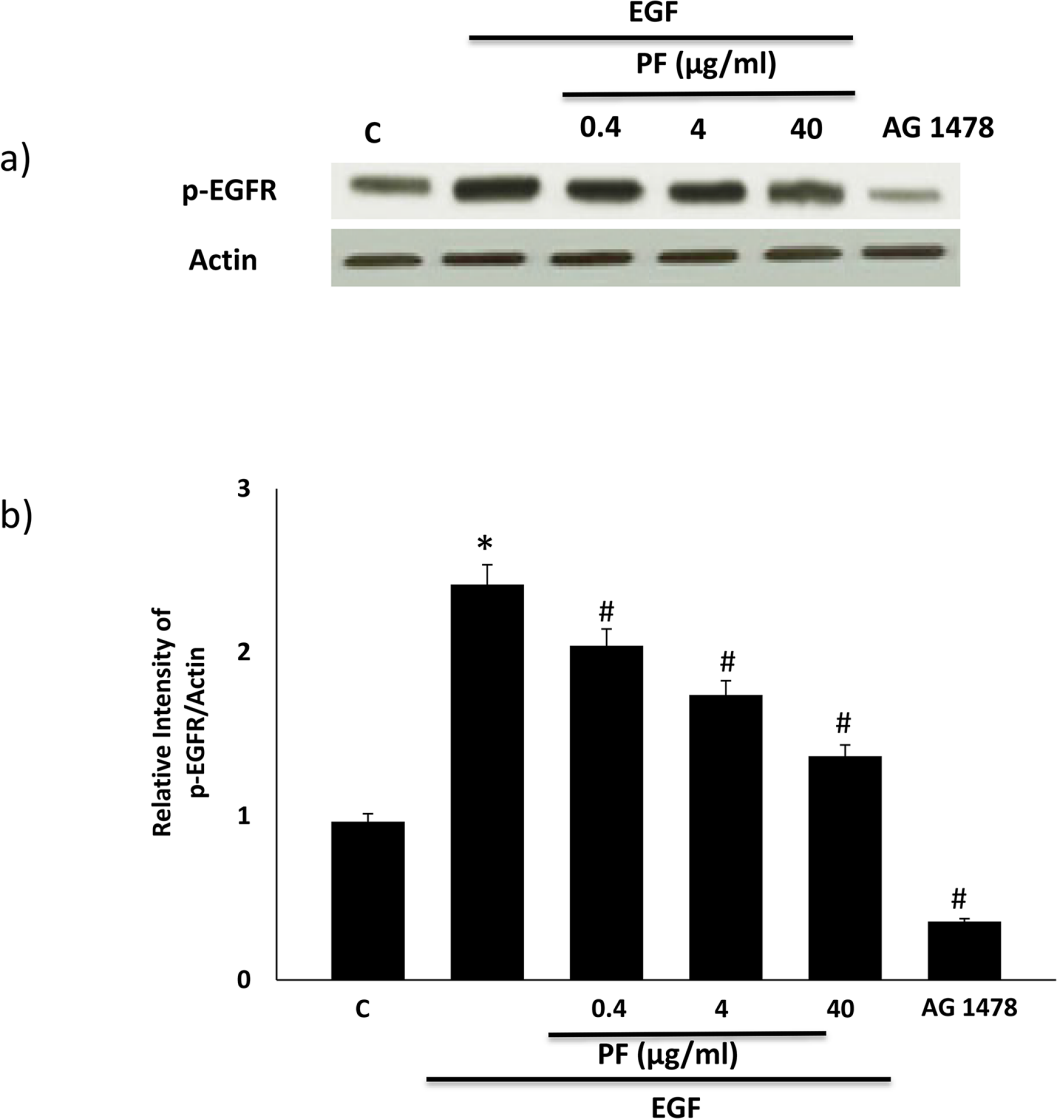
PF PAMAM dendrimer dose-dependently inhibits EGF-ligand induced phosphorylation of EGFR in HEK 293 cells. Cells were pre-treated with the stated doses of PF for 4h or with AG1478 (10 μM) for 24h or treated with EGF alone (10nM) for 30 mins as per the Methods. For PF + EGF co-administration, EGF was only added for the final 30 min prior to cell lysis at 4h. Panel a) shows a representative Western blot and panel b) shows the densitometric histograms showing the relative intensity levels of p-EGFR bands relative to the actin loading control. Error bars represent mean ± SEM. N = 5. Asterix (*) indicates statistically significant from untreated controls whereas # indicates significantly different from EGF alone treated cells.

We next investigated whether PF inhibited the acute stimulation of EGFR phosphorylation by its ligand. Administration of EGF (10nM) led to a robust and reproducible elevation in EGFR phosphorylation (Fig 3) that could be blocked by AG1478, a known inhibitor of EGFR phosphorylation. Increasing doses of PF resulted in a significant reduction in EGF-induced EGFR phosphorylation (p<0.05) but this was to a lesser extent than observed with AG1478 (Fig 3).

### PF dendrimer-mediated inhibition of EGFR phosphorylation is accompanied by inhibition of downstream signaling via ERK1/2 and p38 MAPK

**Fig 4:**
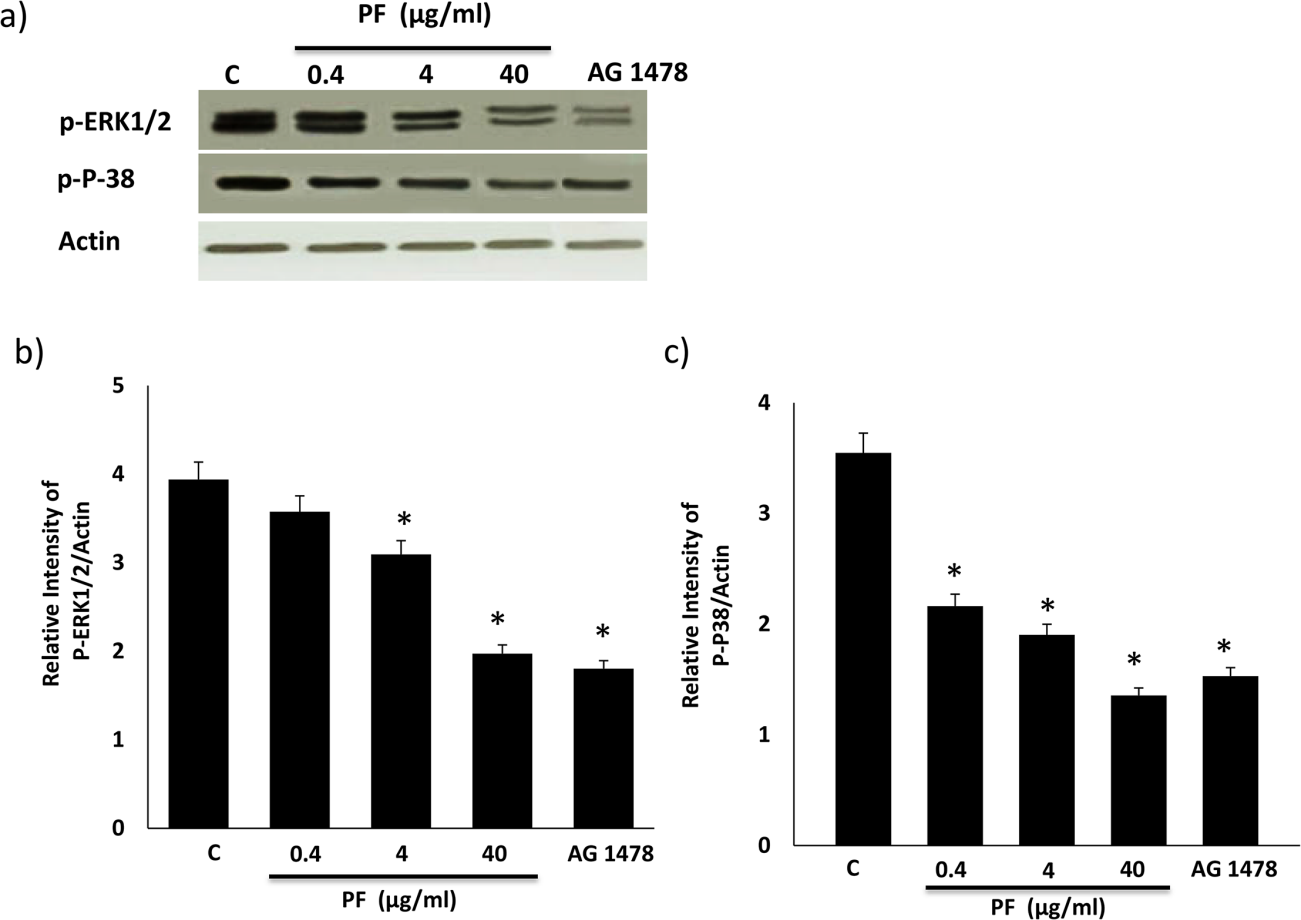
Effects of Polyfect (PF) PAMAM dendrimer dose on phosphorylation of ERK1/2 and p38 MAPK in HEK293 cells. Cells were treated with the stated doses of PF for 4h and analyzed after 24h or with AG1478 for 24h as per the Methods. Panel a) shows representative Western blots and panels b) shows the densitometric histograms showing the relative intensity levels of phosphorylated (p-) ERK1/2 and phosphorylated p38 MAPK (labelled as p-P-38) bands relative to the actin loading control. Error bars represent mean ± SEM. N = 5. * indicates statistically significant from untreated controls.

We next examined whether the effects of PF dendrimer on EGFR phosphorylation were transmitted to its downstream signaling effectors, ERK1/2 and p38 MAPK and also assessed the effects of AG1478, a selective inhibitor of EGFR tyrosine kinase in HEK 293 cells (Fig 4). PF dose-dependently decreased phosphorylation of both ERK1/2 and p38 MAPK which at the 40 μg/ml dose appeared similar to that observed with AG1478 (Fig 4).

### Time-dependent effects of Polyfect PAMAM dendrimer on EGFR phosphorylation

**Fig 5:**
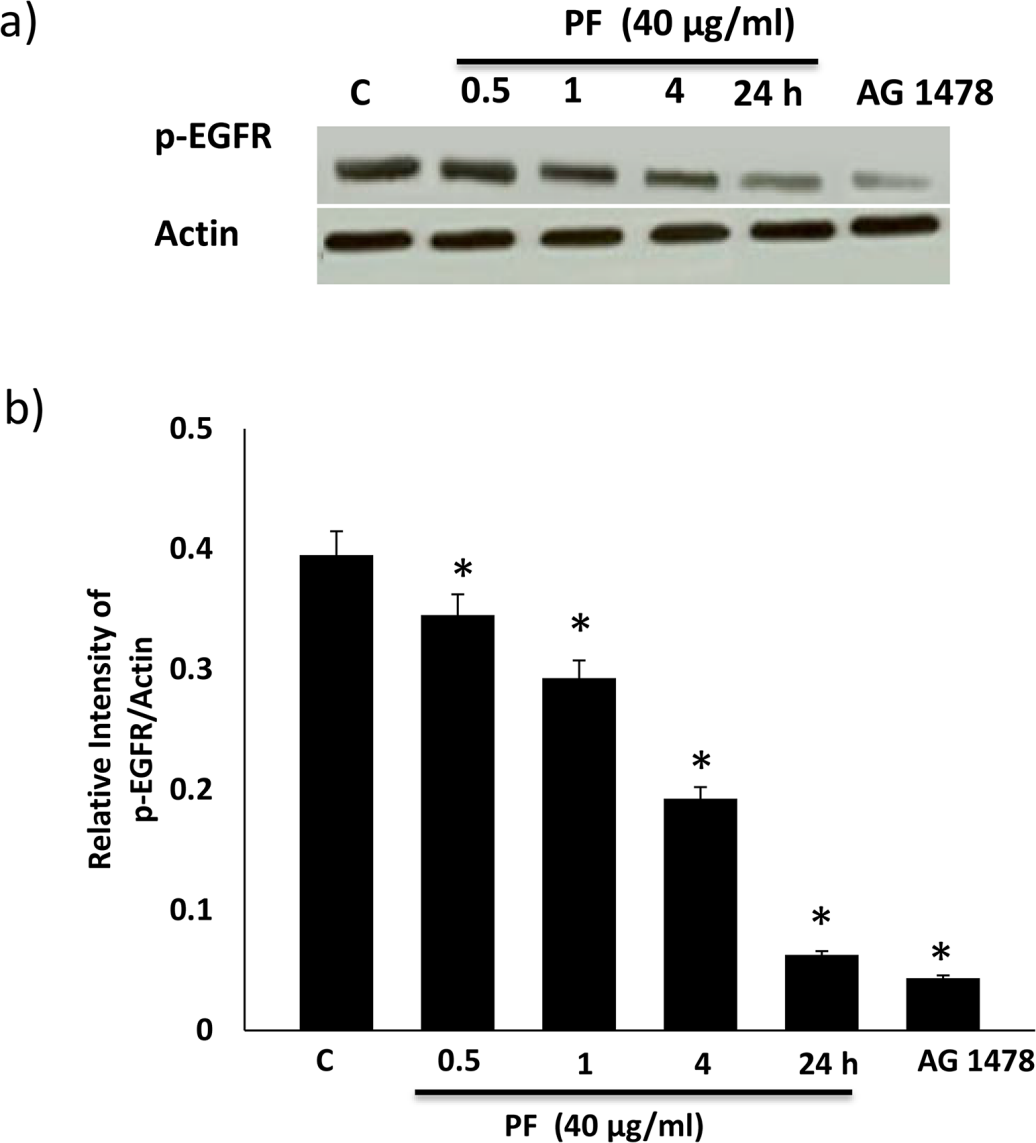
Polyfect (PF) PAMAM dendrimer-mediated inhibition of EGFR phosphorylation as a function of time. HEK 293 cells were treated with SF (40 μg/ml) continuously for the stated time or with AG1478 (10 μM) for 24h. Panel a) shows a representative Western blots and panels b) shows the densitometric histograms showing the relative intensity levels of phosphorylated (p-) EGFR bands relative to the actin loading control. Error bars represent mean ± SEM. * indicates statistically significant from untreated controls. N = 5.

At the 40 μg/ml dose, we next studied the effects of incubation time on Polyfect PAMAM dendrimer-mediated changes in EGFR phosphorylation in HEK 293 cells. PF exhibited a progressively increasing inhibition of EGFR phosphorylation over the 24h time-period of the study (Fig 5). After 24h, PF treatment resulted in a robust and statistically significant inhibition in EGFR phosphorylation (p<0.05; Fig 5) that was similar to that observed with AG1478 (Fig 5).

### *In vivo* studies with Polyfect and Superfect PAMAM dendrimers in normal and diabetic rats

#### i) Effect of PAMAM dendrimers on body weight and blood glucose levels

We next compared the *in vivo* effects of PAMAM dendrimers in normal (non-diabetic) and diabetic rats. As expected diabetes led to a significantly decreased body weight and significantly elevated blood glucose levels (p<0.05;Table 1). However, the 24h acute *in vivo* treatments with PAMAM dendrimers or AG1478 had no significant effect on the body weights or blood glucose levels in either normal or rats made diabetic with streptozotocin (Table 1).

**Table 1:** The effect of AG1478 or PF and SF PAMAM dendrimers on rat body weight and blood glucose following acute 24 hr *in vivo* administration in non-diabetic controls (C) and diabetic animals (D). (Means ± SEM; N = 4).

Group	Animal weight (g)	Blood Glucose (mmol/L)
C	322.5 ± 19.4	4.93 ± 0.28
C + PF	345.0 ± 12.7	5.00 ± 0.28
C + SF	353.5 ± 13.4	4.65 ± 0.21
C + AG1478	340.0 ± 13.2	4.50 ± 0.20
D	277.3 ± 19.8 (*)	29.00 ± 4.13 (*)
D + PF	264.0 ± 15.9 (*)	30.55 ± 2.49 (*)
D + SF	264.5 ± 9.1 (*)	25.25 ± 2.40 (*)
D + AG1478	272.0 ± 18.5 (*)	33.30 ± 2.12 (*)

Asterix (*) indicates significantly different from controls (p<0.05).

#### ii) Effects of PAMAM dendrimer on renal EGFR phosphorylation and kidney morphology in non-diabetic rats

**Fig 6:**
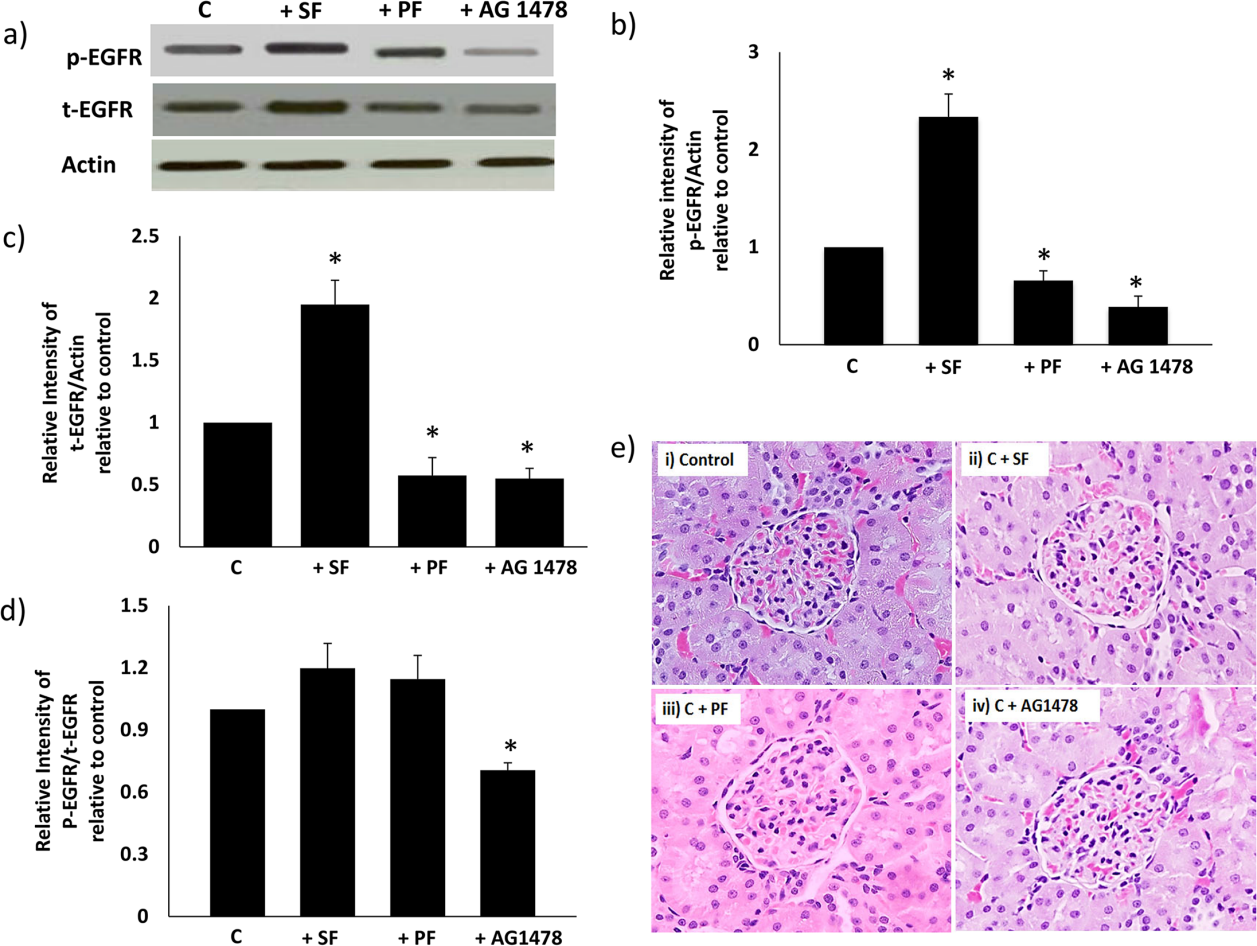
Comparison of the *in vivo* effects of Superfect (SF) and Polyfect (PF) PAMAM dendrimers on EGFR phosphorylation and histopathology in the non-diabetic rat kidney. A single dose of SF or PF (10mg/kg) or AG1478 (1mg/kg) was administered by i.p. injection 24 h prior to sacrifice and subsequent analysis. Panel a) shows representative Western blots and panels b-d) shows the densitometric histograms showing the relative intensity levels of phosphorylated (p-) EGFR bands relative to the actin loading control or to total EGFR protein in the non-diabetic rat kidney. Error bars represent mean ± SEM. N = 4. * indicates statistically significant from untreated controls. Panel e) shows representative photomicrographs of the morphology of the kidneys taken from non-diabetic control (C) animals and those treated with PF or SF or AG1478 as indicated. The kidneys were fixed in formalin and mid-transverse sections taken following paraffin embedding and hematoxylin-eosin staining.

A 24h *in vivo* administration of PF significantly inhibited both EGFR phosphorylation (Fig 6A and 6B) and protein expression (Fig 6A and 6C) in the non-diabetic rat kidney (p<0.05). In contrast, SF led to an increased EGFR phosphorylation (Fig 6A and 6B) and protein expression (Fig 6A and 6C) in the non-diabetic rat kidney. The *in vivo* effects of both dendrimers on EGFR phosphorylation in the non-diabetic kidneys appeared to be fully mirrored by changes in total EGFR protein expression as highlighted by the non-significant changes in the ratio plot of phosphorylated to total EGFR protein compared to controls (Fig 6D). As expected, treatments with AG1478 led to a net reduction in EGFR phosphorylation (Fig 6D) though it too lowered EGFR protein levels but to a lesser extent than phosphorylated EGFR levels (Fig 6A, 6B and 6C).

We next studied the impact of 24h *in vivo* dendrimer treatment on the morphology of kidneys isolated from non-diabetic rats 24h post-treatment. Fig 6E shows that acute *in vivo* treatments with either SF, PF or AG1478 did not alter the gross morphology (as summarized in Table 2) of the non-diabetic kidney.

**Table 2:** The effect of acute *in vivo* administration PF and SF PAMAM dendrimers on morphological changes in non-diabetic and diabetic rat kidneys.

Animal Group	Kidney Morphology^$^				
	GS	EX	FTI	HPAS	IIVCIH
**Control (C)**	0	0	0	0	0
**C + PF**	0	0	0	0	0
**C + SF**	0	0	0	0	0
**C + AG1478**	0	0	0	0	0
**Diabetes (D)**	(++++)	(++++)	(++)	(++++)	(++++)
**D + PF**	(++++)	(++++)	(++)	(++++)	(++++)
**D + SF**	(++++)	(++++)	(++)	(++++)	(++++)
**D + AG1478**	(++++)	(++++)	(++)	(++++)	(++++)

**Key:** Morphology was graded as either (0) none, (+) mild, (++) moderate or (++++) severe.

^**$**^GS: diffuse glomerular sclerosis; EX: extracelluar matrix deposition (or interstitial fibrosis); FTI: fibrosis in tubulointerstitium; HPAS: hyperplastic arteriolosclerosis; IIVCIH: intensity and incidence of vacuolations, cellular infiltration and hypertrophy.

#### iii) Effects of PAMAM dendrimer on renal EGFR phosphorylation and kidney morphology in diabetic rats

**Fig 7:**
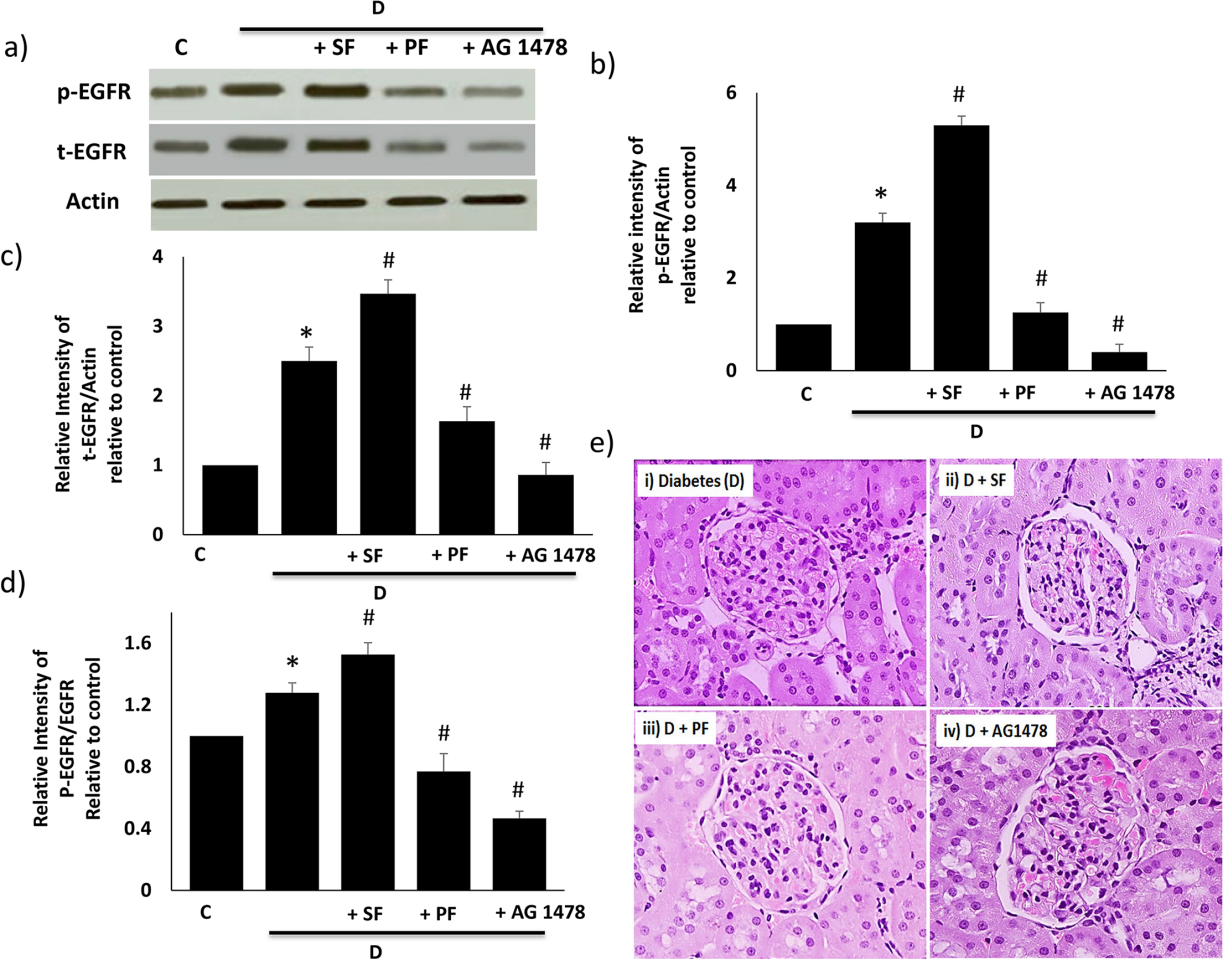
The *in vivo* effects of Superfect (SF) and Polyfect (PF) PAMAM dendrimers on EGFR phosphorylation and histopathology in the kidney of diabetic rats. After 4 weeks of diabetes, a single dose of SF or PF (10mg/kg) or AG1478 (1mg/kg) was administered by i.p. injection 24 h prior to sacrifice and subsequent analysis. Panel a) shows representative Western blots and panels b-d) shows the densitometric histograms showing the relative intensity levels of phosphorylated (p-) EGFR bands relative to the actin loading control or to total EGFR protein in the non-diabetic rat kidney. Error bars represent mean ± SEM. N = 4. * indicates statistically significant from untreated controls and # indicates statistically significant from diabetes. Panel e) shows representative photomicrographs of the morphology of the kidneys taken from diabetic control (D) animals and those treated with PF or SF or AG1478 as indicated. The kidneys were fixed in formalin and mid-transverse sections taken following paraffin embedding and hematoxylin-eosin staining.

We also examined the *in vivo* effects of PAMAM dendrimers on the kidneys isolated from diabetic rats as these provided a model with a higher basal EGFR activity [41,42]. Diabetes resulted in a net increased EGFR phosphorylation in the diabetic kidney compared to control animals (Fig 7A and 7D). A 24h administration of Polyfect to diabetic rats led to a significant reduction in EGFR phosphorylation and in total EGFR (Fig 7A–7C). In contrast, SF led to a net stimulation of EGFR phosphorylation that was also accompanied by an increase in total EGFR protein (Fig 7A–7D). In the diabetic kidney, both dendrimers appeared to have a net greater effect on EGFR phosphorylation than on protein expression as highlighted by the statistically significant changes in the ratio plot of phosphorylated to total EGFR protein compared to controls (p<0.05) (Fig 7D). Relative to non-diabetic control kidneys, there was a marked increase in pathological abnormalities observed in the diabetic kidney such as diffuse glomerular sclerosis, extracellular matrix deposition, tubulointerstitial fibrosis and increased incidence of vacuolations, cell infiltration and hypertrophy (see Table 2). However, the acute 24h treatments with PF, SF or AG1478 did not alter the kidney morphology from that observed in diabetes alone (Fig 7E; Table 2).

### Dendrimer-mediated changes in EGFR-ERK1/2-p38 MAPK signaling can be attenuated by antioxidants in HEK 293 cells

**Fig 8:**
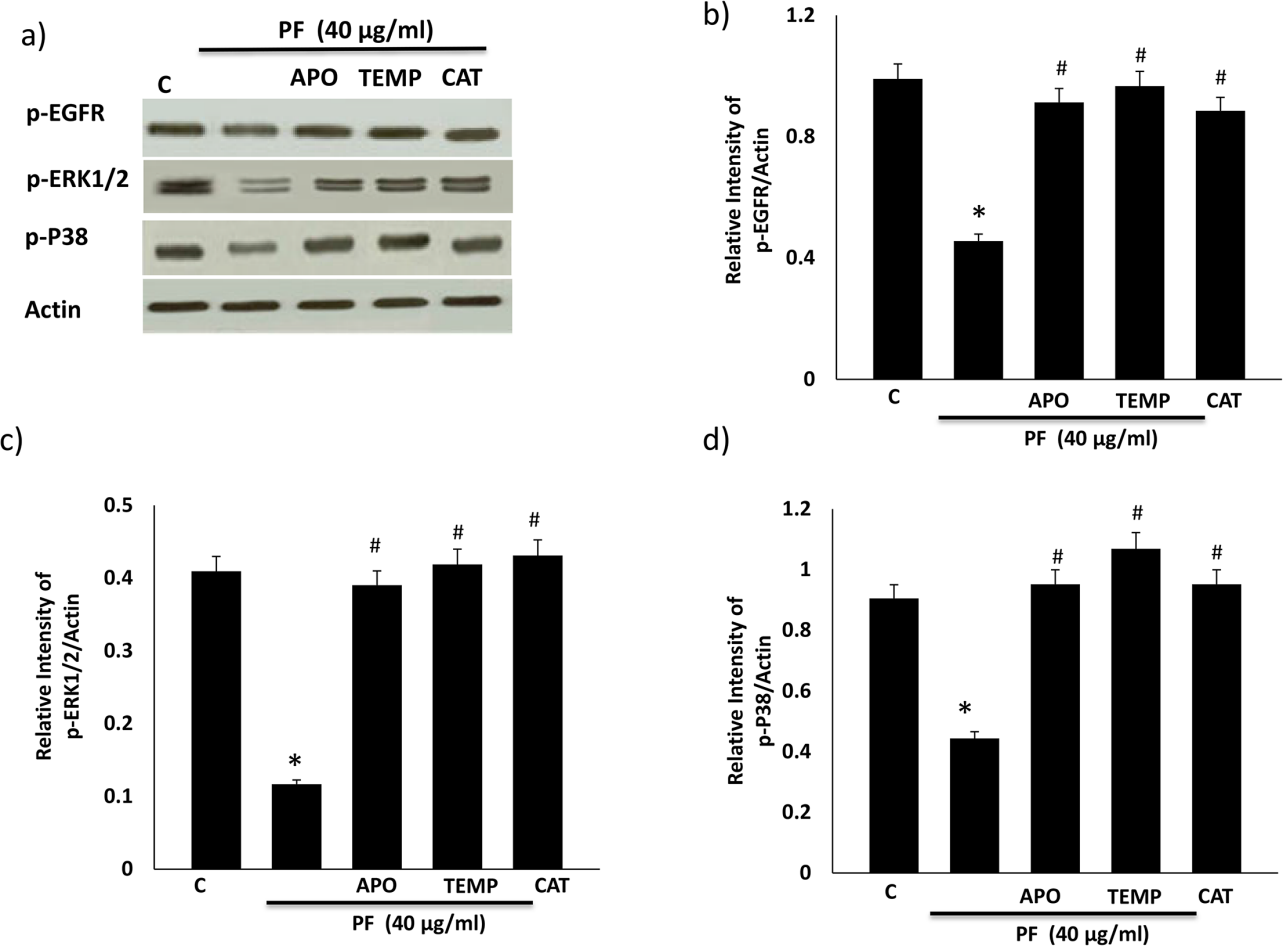
The effect of anti-oxidants apocynin (APO), Tempol (TEMP) and catalase (CAT) on PF PAMAM dendrimer-induced EGFR, ERK1/2 and p38 MAPK phosphorylation in HEK 293 cells. Cells were pretreated for 30 min with the stated antioxidant and then co-treated with PF (40 μg/ml) for 4h in serum-free media. Panel a) shows a representative Western blot and panel b-d) shows the densitometric histograms showing the relative intensity levels of phosphorylated (p-) EGFR, ERK1/2 and p38 MAPK (labelled as p-P38) bands relative to the actin loading control. Error bars represent mean ± SEM. N = 5. * indicates statistically significant from untreated controls # indicates statistically significant from PF alone treatment.

To further study the cellular mechanisms involved in modulation of EGFR signaling with PF PAMAM dendrimer, we investigated the role of oxidative stress through the use of antioxidants. Thus, we examined the effects of pretreating HEK 293 cells with well-known antioxidants apocynin, catalase and tempol prior to exposing cells to PF PAMAM dendrimers and subsequent analysis of EGFR, ERK1/2 and p38 MAPK phosphorylation. Apocynin, catalase and tempol, at doses previously shown to prevent elevation of EGFR phosphorylation induced by the well-known oxidative stress mimetic, hydrogen-peroxide [36], also significantly attenuated PF-induced elevation in EGFR, ERK1/2 and p38 MAPK phosphorylation (p<0.05; Fig 8).

### PF and SF PAMAM dendrimer-mediated apoptosis occurs via an oxidative stress-dependent mechanism

**Fig 9:**
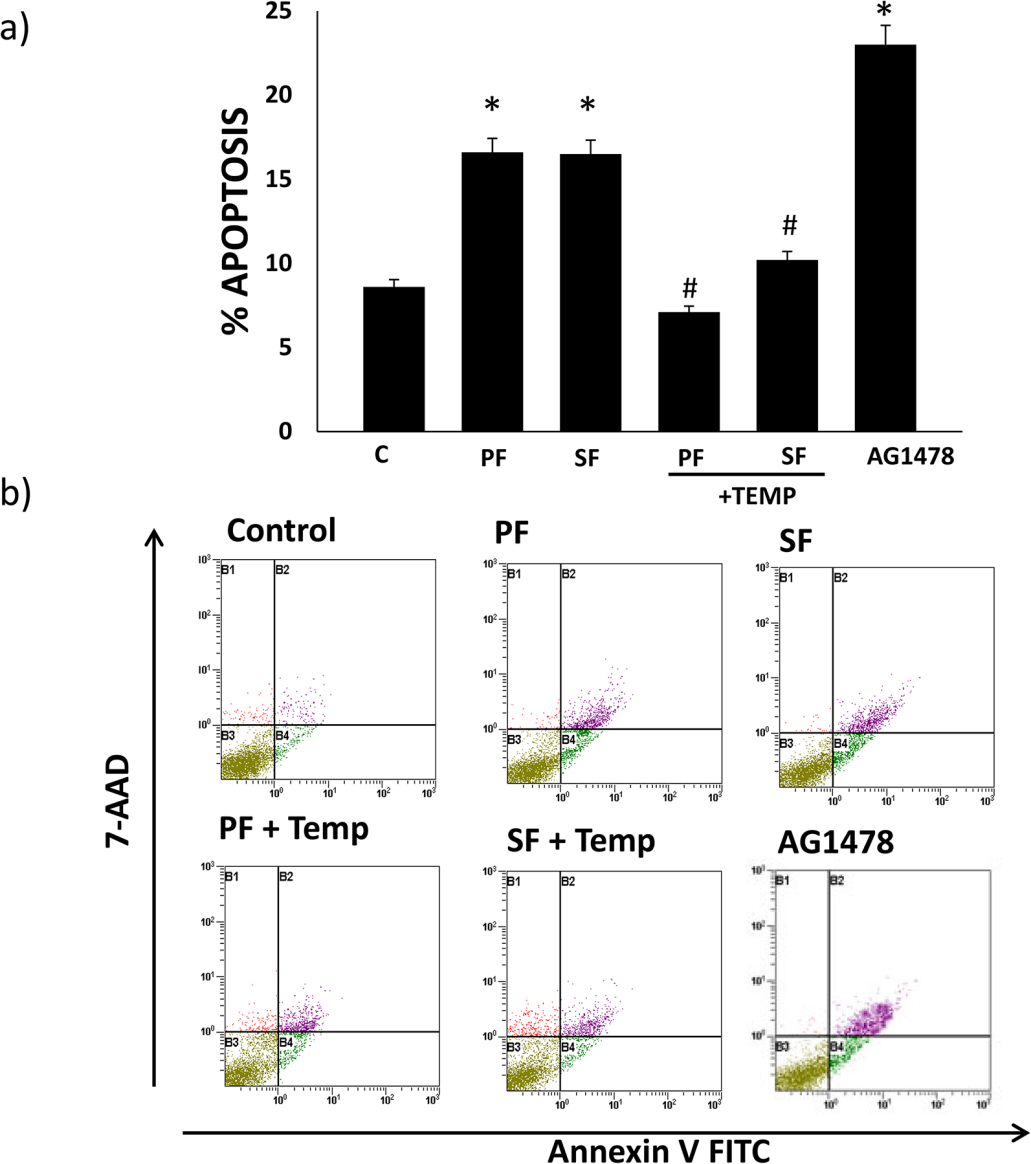
Superfect (SF) and Polyfect (PF) PAMAM dendrimer-mediated apoptosis is inhibited by the antioxidant, Tempol, in HEK293 cells. The % of cells undergoing apoptosis is shown as a) histograms and b) representative flow cytometry data plots for cells treated with PF alone, SF alone or together with Tempol (+ TEMP) and AG1478 alone. Error bars represent mean ± SEM. N = 5. * indicates statistically significant from control cells # indicates statistically significant from the respective dendrimer alone treatment.

We next investigated whether PF and SF PAMAM dendrimers induced cell death by apoptosis and whether this was sensitive to antioxidant treatment. Both PF and SF treatment led to increased apoptosis in HEK 293 cells to a similar extent as assessed by flow cytometry (Fig 9). The antioxidant, tempol, significantly reduced dendrimer-mediated apoptosis for both PF and SF. AG1478, at a 10-fold higher dose (100 μM) than used in signaling studies, was used as a positive control and significantly induced apoptosis in HEK 293 cells (Fig 9).

## Discussion

This study highlights a novel biological action of PAMAM dendrimers in that non-activated and activated G6 PAMAM dendrimers, Polyfect and Superfect respectively, can differentially modulate EGFR signaling *in vivo*. Thus our data imply that these dendritic polymers might be considered as a novel class of modulators of EGFR phosphorylation that can be selected to either stimulate or inhibit EGFR signaling- an important cellular signal transduction pathway in diseases such as cancer and diabetes-induced cardiovascular and renal diseases. In two animal models, these PAMAM dendrimer-mediated changes in EGFR signaling appear to precede any significant changes in renal morphology over the 24h study period. Thus, implying that acute *in vivo* exposures to dendrimers can have a marked effect on signal transduction pathways in the absence of any overt toxicity as assessed by morphological changes in the kidneys. Further, PAMAM dendrimers appear to modulate EGFR signaling and apoptosis through an oxidative stress-dependent mechanism. To the best of our knowledge this study shows for the first time that PAMAM dendrimers can modulate the important EGFR signal transduction pathway both *in vitro* and *in vivo* and may have important clinical implications for their use in nanomedicine.

It is now become increasingly clear that cationic PAMAM dendrimers exhibit a range of biological and toxicological actions that need to be fully understood for their safe use in (nano) medical applications. In an attempt to understand their biological interactions at the level of signal transduction pathways, we have been investigating their effects on the highly important EGFR signal transduction pathway. We previously showed that the activated PAMAM dendrimer, SF, was a robust inducer of EGFR phosphorylation and downstream signaling via ERK1/2 in HEK 293 cells [36]. Here, we show that PF has the opposing effect in that it dose-dependently inhibits basal and EGF-ligand induced EGFR phosphorylation (Figs 2 and 3) as well as downstream signaling via ERK1/2 and p38 MAPK in HEK 293 cells (Fig 4). It should be noted that at the 40 μg/ml concentration, PF-mediated reduction in EGFR protein (see Fig 2C) might also partly account for the attenuation in downstream signaling activity. The inhibition of EGFR phosphorylation appears to be robust and sustained as it was observed over a 24h time period with PF PAMAM dendrimer in HEK 293 cells (Fig 5). Thus, PF studied here and SF, reported on previously in the same cells [36], appear to have opposing effects of EGFR signaling in that PF acts an inhibitor whereas as SF behaves as a stimulator or activator of EGFR phosphorylation and signaling in HEK 293 cells. These effects are likely to be general and not specific to HEK 293 cells–a notion supported by findings in cultured A431 cells where SF and PF appear to have opposing effects on EGFR mRNA and protein expression that ultimately differentially affect the efficacy of siRNA delivered by these PAMAM dendrimers [18]. It is noteworthy, that PF-induced modulation of EGFR-ERK1/2-p38 MAPK occurred at doses that only showed little or no overt cytotoxicity (at up to 4 μg/ml dose) and with only about 20% reduction in cell viability at the highest 40 μg/ml dose studied (Fig 1).

**Fig 10:**
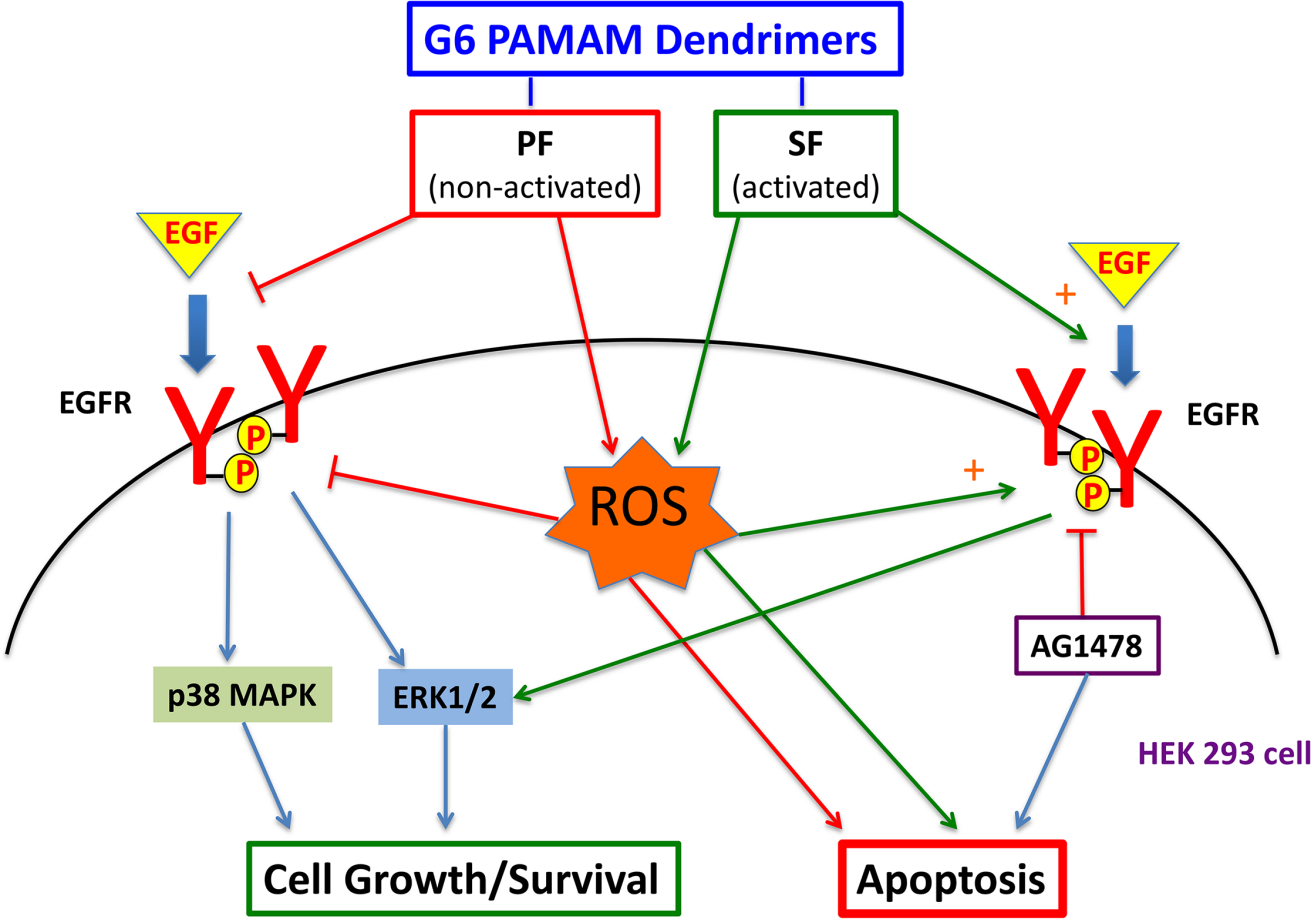
A schematic diagram highlighting our current understanding of the differential effects of PF and SF PAMAM dendrimers on EGFR-ERK1/2-p38MAPK signaling and apoptosis via an oxidative-dependent mechanism in HEK 293 cells. Data from the present study and from Akhtar et al [36] are summarized. We previously showed that SF stimulated basal EGFR phosphorylation via an oxidative-stress dependent mechanism and also enhanced EGF-ligand induced activation (Akhtar et al, 2013). In the present study, we showed that PF also inhibits basal EGFR phosphorylation via an oxidative stress mechanism as well as EGF-ligand induced activation of EGFR. SF and PF both induced apoptosis via an oxidative-stress/ROS dependent mechanism. AG1478, a selective inhibitor of EGFR tyrosine kinase, inhibited the stimulatory effects of SF and also induced apoptosis in HEK 293 cells (Akhtar et al, 2013). Antioxidants inhibiting formation of reactive oxygen species/oxidative stress were able to prevent PF and SF induced modulation of basal EGFR activation and dendrimer-induced apoptosis.

To determine whether these PAMAM dendrimer-mediated effects on EGFR signaling extended beyond the *in vitro* cell culture scenario, we investigated the effects of PF and SF in whole animal studies. Based on pharmacokinetic and biodistribution studies showing significant accumulation of dendrimers in kidneys [2,43,44], we studied their effects on EGFR signaling in the kidneys of two animal models that exhibited low (non-diabetic rats) and high (diabetic rats) basal levels of EGFR expression and phosphorylation (Figs 6 and 7). Following a single dose (10mg/kg) i.p administration of PAMAM dendrimers for a 24 h time period, we noted that in both animal models PF inhibited whereas SF stimulated EGFR protein expression and phosphorylation, though there appeared to be a net effect on phosphorylation by the dendrimers only in the diabetic kidney where there was approximately 2.5-fold higher basal EGFR tyrosine kinase activity. Also, the effect of SF stimulation was greater with an approximately 240% increase in the non-diabetic kidney that had lower basal EGFR activity compared to about a 140% increase in EGFR phosphorylation in the diabetic kidney. Therefore, it is likely that since the levels of phosphorylated EGFR are already very high in the diabetic kidney, SF cannot enhance them much further. However, in the non-diabetic kidney where EGFR basal activity is quite low, a much higher SF-induced stimulation in EGFR phosphorylation was observed. PF appeared to inhibit renal EGFR phosphorylation in both sets of animals to a similar degree in the range of 40–50% inhibition following the 24h administration. Taken together our data from two animal models as well as the *in vitro* cell culture studies presented here and previously [36] confirm that SF and PF have opposing effects on EGFR signaling (for a summary schematic see Fig 10). Though further studies are needed, this leads us to speculate that PAMAM dendrimers may represent a novel class of EGFR modulators that may serve as alternatives to those already available such as the small molecule tyrosine kinase inhibitors, receptor ligands and monoclonal antibodies and for which cells can develop resistance mechanisms [45,46].

Since we previously showed that EGFR activation by either hydrogen peroxide (H_2_O_2_) or SF occurred via an induction of reactive oxygen species (ROS)/ oxidative-stress dependent mechanism [36], we also investigated whether PF-mediated modulation of EGFR signaling was occurring via a similar mechanism and was sensitive to treatment with anti-oxidants in HEK 293 cells. Interestingly, at concentrations that significantly reduced SF or H_2_O_2_-induced elevation in EGFR phosphorylation previously [36], the antioxidants, apocynin (an inhibitor of NADPH oxidase activity that leads to cellular generation of reactive oxygen species such as superoxide [47], tempol (a superoxide dismutase (SOD) mimetic and free radical scavenger [48] and catalase (a redox enzyme that catalyzes the decomposition of hydrogen peroxide to water and oxygen [47] were also able to significantly attenuate PF PAMAM dendrimer-mediated inhibition of EGFR, ERK1/2 and p38 MAPK phosphorylation in HEK 293 cells. Surprisingly, these data suggest that inhibitory effects of the non-activated PF dendrimer on EGFR signaling, as well as the previoulsy studied stimulatory effects of the activated SF [36], are both mediated by PAMAM dendrimer-induced oxidative stress in HEK293 cells. As to how exactly oxidative-stress or reactive oxygen species (ROS) generation can lead to such opposing effects on EGFR signaling is unclear and requires further study.

Importantly, although our studies suggest that PAMAM-induced oxidative stress presumably after cellular internalization leads to modulation of EGFR signaling, it does not rule out direct interactions of dendrimers at the level of EGFR receptor or through modulation of upstream and/or downstream effectors and subsequent impact via negative or positive feedback loops. For example, we have previously shown that the differential effects of PF and SF may be explained by the opposing effects on EGFR gene expression in the nucleus [18]. Nonetheless, since we observed that PF significantly attenuated the stimulatory effects of EGF-ligand on EGFR phosphorylation (Fig 3), it is conceivable that this can occur via inhibition of EGF-ligand binding in the classical ligand-mediated activation of EGFR (see also Fig 10). Alternatively, PAMAM dendrimers may interfere with other upstream activators of EGFR such as G-protein coupled receptors that induce EGFR phosphorylation via src- or metalloproteinase-dependent mechanisms [49]. Another potential site for modulation of EGFR phopshorylation would be the ATP-binding site on the intracellular tyrosine kinase to which PF and SF might conceivably interfere differently to either induce or inhibit ATP binding and EGFR phosphorylation. Recent studies suggest that to avoid ligand-independent receptor activation, the intracellular tyrosine kinases of monomeric and dimeric forms of EGFR are thought to electrostatically dock via their positively charged amino acid residues onto negatively charged lipids on the inside of the plasma membrane to avoid transphosphorylation during receptor autoinhibiton [31,50–52]. Since cationic PAMAM dendrimers are known to electrostatically bind with negatively charged cell-surface membrane lipids and to produce nanosized cell-membrane pores [9] or even ion-channels [21] leads us to speculate that surface-bound or internalized PAMAM polymers via their positive charges could readily disrupt the electrostatic interactions between the receptor tyrosine kinase domains and plasma membrane. Since differences in charge and/or branching density can account for differential effects with PAMAM dendrimers [53], the opposing effects on EGFR signaling with non-activated PF (inflexible dense hyperbranched archtecture) versus the activated SF (flexible nature with reduced internal branching) could conceivably arise from differential interactions of these two PAMAM dendrimers at the level of receptor autoinhibition, though these need to be studied in detail. Alternatively since EGFR signaling is reported to occur within endosomes [54,55], PAMAM dendrimers might also differentially alter EGFR signaling during its known uptake via clathrin-coated endocytosis [56,57] and co-residence within these sub-cellular organelles.

Since EGFR inhibition can lead to cell growth inhibition and cell death by apoptosis [28,58] and the fact that apoptosis is one of the cellular toxicity mechanisms reported for PAMAM dendrimers [59], we also studied the effect of PF and SF PAMAM dendrimers on apoptosis in HEK 293 cells in the presence of the anti-oxidant, tempol. Surprisingly, both PF and SF PAMAM dendrimers significantly induced apoptosis in HEK 293 cells in a manner similar to AG1478 implying that, at least in the case of SF which stimulated EGFR phosphorylation, dendrimer-mediated apoptosis was independent of its effects on EGFR signaling. Both PF- and SF-mediated apoptosis could be reversed by tempol, implying that rather like dendrimer-induced modulation of EGFR signaling, dendrimer-induced apoptosis also occurs via an oxidative-stress dependent mechanism for both the activated and non-activated PAMAMs. Indeed, the generation of reactive oxygen species as a mechanism of nanoparticle-induced toxicity in general has been reported previously [60–62]. Thus, our study suggests that oxidative stress or ROS generation appears a common mechanism through which SF and PF induce apoptosis and differentially regulate EGFR signaling. As to how exactly oxidative stress leads to these differential effects and how PAMAM dendrimer-induced oxidative stress and apoptosis might impact cell/organ function *in vivo* requires further study.

End-organ nephropathy and renal damage are commonly observed in diabetes that in some cases can be linked to increased EGFR expression and activity as well as oxidative stress [41,42,63]. In animals bearing 4 weeks of diabetes significant pathology such as diffuse glomerular sclerosis, extracellular matrix deposition, tubulointerstitial fibrosis and increased incidence of vacuolations, cell infiltration and hypertrophy was observed (see Figs 6 and 7; Table 2) and was similar to that previously described in other studies [40,41]. However, acute 24h treatments with either PAMAM dendrimer or AG1478 did not alter the morphology of kidneys isolated from diabetic or non-diabetic animals nor lead to any significant changes in animal weights or blood glucose levels. Our findings are supported by literature reports showing that EGFR-induced renal pathologies such as hypertrophy, glomerular enlargement and fibrosis can only be corrected by chronic treatments with EGFR inhibitors [41,42,64]. For example, a recent study in a model of type 1 diabetes showed that up to 24 weeks of treatment with the EGFR inhibitor, Erlinotib, attenuated progression of diabetic nephropathy [41]. Similarly the hypoglycemic effect associated with G4 PAMAM dendrimers was noted upon chronic daily treatment for up to 60 days [22]. Thus, further *in vivo* studies are needed whereby SF and PF are administered chronically over several weeks or months to fully investigate their possible opposing impact on diabetes-induced kidney damage. Such studies are planned.

The data presented here also lend further support to the assertion that drug delivery systems cannot be considered as biologically inert and aside from their ability to improve drug delivery have additional, sometimes unexpected, biological and cellular effects [15–17,65]. Our study shows that *in vivo* modulation of the highly important EGFR cell signal transduction pathway can also be added to the growing list of intrinsic biological activities for PAMAM dendrimers that includes eliciting multiple gene expression changes in biological systems that may impact on siRNA activity and specificity [18,66], disrupting key platelet function [67], modulating Angiotensin-converting enzyme 2 activity [68] and exhibiting anti-inflammatory activity [20,38].

In conclusion, to the best of our knowledge this is the first study showing that activated and non-activated PAMAM dendrimers can differentially interfere with the important EGFR cell signal transduction pathway *in vivo* at doses that do not lead to overt toxicity as assessed by gross morphological changes in the kidney. Although the exact long-term consequences of activating cellular signal transduction pathways such as EGFR-ERK1/2-p38 MAPK by PAMAM dendrimers *in vivo* remain unknown, our data presented here provides a novel insight into the differential protein interactions of activated and non-activated G6 PAMAM dendrimers and underscores the necessity for further research into the biological activity and toxicology of these nanosystems prior to their use in clinical applications. Further, the fact that PAMAM dendrimers can either stimulate or inhibit EGFR phosphorylation *in vitro* and *in vivo* might lead to their use as novel modulators of EGFR activity and signaling that could offer novel opportunities for therapeutic intervention especially in diabetes-induced end organ pathologies. For example, PF might be useful in reversing diabetes-induced renal and vascular dysfunction where overactivity of EGFR is noted [33,40–42,69]. Alternatively, SF might be used in place of conventional ligand-activation based therapeutic strategies to stimulate EGFR signaling in diabetes-induced cardiac dysfunction which results from depressed EGFR-ERK1/2 phosphorylation [34]. However, due to the differential regulation of EGFR in tissues such as the heart and vasculature, it is likely that targeted organ-specific delivery of PAMAMs will be required to achieve the desired pharmacological effects.

## References

[1] Duncan R, Izzo L. Dendrimer biocompatibility and toxicity. Adv. Drug Deliv. 2005;57: 2215–2237. 10.1016/j.addr.2005.09.019 16297497

[2] Labieniec-Watala M, Watala C. PAMAM dendrimers: destined for success or doomed to fail? Plain and modified PAMAM dendrimers in the context of biomedical applications. J Pharm Sci. 2015;104(1): 2–14. 10.1002/jps.24222 25363074

[3] Pettit MW, Griffiths P, Ferruti P, Richardson SC. Poly(amidoamine) polymers: soluble linear amphiphilic drug-delivery systems for genes, proteins and oligonucleotides. Ther. Deliv. 2011;2: 907–917. 22833902 10.4155/tde.11.55

[4] Tomalia DA, Reyna LA, Svenson S. Dendrimers as multi-purpose nanodevices for oncology drug delivery and diagnostic imaging. Biochem. Soc. Trans. 2007;35: 61–67. 17233602 10.1042/BST0350061

[5] Shakhbazau A, Isayenka I, Kartel N, Goncharova N, Seviaryn I, Kosmacheva S. Transfection efficiencies of PAMAM dendrimers correlate inversely with their hydrophobicity. Int J Pharm. 2010;383: 228–235. 10.1016/j.ijpharm.2009.09.020 19770028

[6] Tang MX, Szoka FC. The influence of polymer structure on the interactions of cationic polymers with DNA and morphology of the resulting complexes. Gene Ther. 1997;4: 823–832. 9338011 10.1038/sj.gt.3300454

[7] Tang MX, Redemann CT, Szoka FC Jr. In vitro gene delivery by degraded polyamidoamine dendrimers. Bioconjug. Chem. 1996;7: 703–714. 8950489 10.1021/bc9600630

[8] Luo K, He B, Wu Y, Shen Y, Gu Z. Functional and biodegradable dendritic macromolecules with controlled architectures as nontoxic and efficient nanoscale gene vectors. Biotechnol Adv. 2014;32(4): 818–830. 10.1016/j.biotechadv.2013.12.008 24389086

[9] Hong S, Leroueil PR, Janus EK, Peters JL, Kober MM, Islam MT, et al Interaction of polycationic polymers with supported lipid bilayers and cells: nanoscale hole formation and enhanced membrane permeability. Bioconjug Chem. 2006;17(3): 728–734. 16704211 10.1021/bc060077y

[10] Sadekar S, Ghandehari H. Transepithelial transport and toxicity of PAMAM dendrimers: Implications for oral drug delivery. Adv. Drug Deliv. 2012;64: 571–588. 10.1016/j.addr.2011.09.010 PMC3305851 21983078

[11] Conti DS, Brewer D, Grashik J, Avasarala S, da Rocha SR. Poly(amidoamine) dendrimer nanocarriers and their aerosol formulations for siRNA delivery to the lung epithelium. Mol Pharm. 2014;11(6): 1808–1822. 10.1021/mp4006358 24811243 PMC4051247

[12] Shcharbin D, Shakhbazau A, Bryszewska M. Poly(amidoamine) dendrimer complexes as a platform for gene delivery. Expert Opin Drug Deliv. 2013;10(12): 1687–1698. 10.1517/17425247.2013.853661 24168461

[13] Zhang Y, Zhou C, Kwak KJ, Wang X, Yung B, Lee LJ, et al Efficient siRNA delivery using a polyamidoamine dendrimer with a modified pentaerythritol core. Pharm Res. 2012;29: 1627–1636. 10.1007/s11095-012-0676-x 22274556 PMC4289905

[14] Movassaghian S, Moghimi HR, Shirazi FH, Torchilin VP. Dendrosome-dendriplex inside liposomes: as a gene delivery system. J. Drug Target. 2011;19: 925–932. 10.3109/1061186X.2011.628396 22023509

[15] Akhtar S. Cationic nanosystems for the delivery of small interfering ribonucleic acid therapeutics: a focus on toxicogenomics. Expert Opin Drug Metab Toxicol. 2010;6: 1347–1362. 10.1517/17425255.2010.518611 20929276

[16] Akhtar S, Benter IF. Nonviral delivery of synthetic siRNAs in vivo. J. Clin. Invest. 2007a;117: 3623–3632. 18060020 10.1172/JCI33494 PMC2096447

[17] Akhtar S, Benter IF. Toxicogenomics of non-viral drug delivery systems for RNAi: Potential impact on siRNA-mediated gene silencing activity and specificity. Adv. Drug Deliv. 2007b;59: 164–182. 10.1016/j.addr.2007.03.010 17481774

[18] Hollins AJ, Omidi Y, Benter IF, Akhtar S. Toxicogenomics of drug delivery systems: Exploiting delivery system-induced changes in target gene expression to enhance siRNA activity. J. Drug Target. 2007;15: 83–88. 17365277 10.1080/10611860601151860

[19] Akhtar S. Non-viral cancer gene therapy: beyond delivery. Gene Ther. 2006;13(9): 739–740. 18360944 10.1038/sj.gt.3302692

[20] Tang Y, Han Y, Liu L, Shen W, Zhang H, Wang Y, et al Protective effects and mechanisms of G5 PAMAM dendrimers against acute pancreatitis induced by caerulein in mice. Biomacromolecules. 2015;16(1): 174–182. 10.1021/bm501390d 25479110

[21] Nyitrai G, Keszthelyi T, Bóta A, Simon A, Tőke O, Horváth G, et al Sodium selective ion channel formation in living cell membranes by polyamidoamine dendrimer. Biochim Biophys Acta. 2013;1828(8): 1873–1880. 10.1016/j.bbamem.2013.04.004 23597947

[22] Labieniec-Watala M, Przygodzki T, Sebekova K, Watala C. Can metabolic impairments in experimental diabetes be cured with poly(amido)amine (PAMAM) G4 dendrimers? In the search for minimizing of the adverse effects of PAMAM administration. Int J Pharm. 2014;464(1–2): 152–167. 10.1016/j.ijpharm.2014.01.011 24463003

[23] Labieniec M, Watala C. Use of poly(amido)amine dendrimers in prevention of early non-enzymatic modifications of biomacromolecules. Biochimie. 2010;92: 1296–1305. 10.1016/j.biochi.2010.06.002 20542077

[24] Labieniec M, Ulicna O, Vancova O, Glowacki R, Sebekova K, Bald E, et al PAMAM G4 dendrimers lower high glucose but do not improve reduced survival in diabetic rats. Int. J. Pharm. 2008;364: 142–149. 10.1016/j.ijpharm.2008.08.001 18761397

[25] Förstner P, Bayer F, Kalu N, Felsen S, Förtsch C, Aloufi A, et al Cationic PAMAM dendrimers as pore-blocking binary toxin inhibitors. Biomacromolecules. 2014;15(7): 2461–2474. 10.1021/bm500328v 24954629 PMC4215879

[26] Durán-Lara E, Guzmán L, John A, Fuentes E, Alarcón M, Palomo I, et al PAMAM dendrimer derivatives as a potential drug for antithrombotic therapy. Eur J Med Chem. 2013;69: 601–608. 10.1016/j.ejmech.2013.08.047 24095753

[27] Jain S, Pitoc GA, Holl EK, Zhang Y, Borst L, Leong KW, et al Nucleic acid scavengers inhibit thrombosis without increasing bleeding. Proc. Natl. Acad. Sci. 2012;109: 12938–12943. 10.1073/pnas.1204928109 22837404 PMC3420207

[28] Roskoski R Jr. The ErbB/HER family of protein-tyrosine kinases and cancer. Pharmacol Res. 2014;79: 34–74. 10.1016/j.phrs.2013.11.002 24269963

[29] Dhomen NS, Mariadason J, Tebbutt N, Scott AM. Therapeutic targeting of the epidermal growth factor receptor in human cancer. Crit Rev Oncog. 2012;17: 31–50. 22471663 10.1615/critrevoncog.v17.i1.40

[30] Juliano RL, Carver K, Cao C, Ming X. Receptors, endocytosis, and trafficking: the biological basis of targeted delivery of antisense and siRNA oligonucleotides. J Drug Target. 2012;21(1): 27–43. 23163768 10.3109/1061186X.2012.740674 PMC3712333

[31] Endres NF, Barros T, Cantor AJ, Kuriyan J. Emerging concepts in the regulation of the EGF receptor and other receptor tyrosine kinases. Trends Biochem Sci. 2014;39(10):437–446. 10.1016/j.tibs.2014.08.001 25242369

[32] Siddiqui S, Fang M, Ni B, Lu D, Martin B, Maudsley S. Central role of the EGF receptor in neurometabolic aging. Int. J. Endocrinol. 2012; 2012: 739428 10.1155/2012/739428 22754566 PMC3382947

[33] Akhtar S, Yousif MH, Dhaunsi GS, Sarkhouh F, Chandrasekhar B, Attur S et al Activation of ErbB2 and Downstream Signaling via Rho Kinases and ERK1/2 Contributes to Diabetes-Induced Vascular Dysfunction. PLoS One. 2013a;8: e67813. 23826343 10.1371/journal.pone.0067813 PMC3694874

[34] Akhtar S, Yousif MH, Chandrasekhar B, Benter IF. Activation of EGFR/ERBB2 via pathways involving ERK1/2, P38 MAPK, AKT and FOXO enhances recovery of diabetic hearts from ischemia-reperfusion injury. PLoS One. 2012a;7(6): e39066. 22720029 10.1371/journal.pone.0039066 PMC3374768

[35] Akhtar S, Yousif MH, Dhaunsi GS, Chandrasekhar B, Al-Farsi O, Benter IF. Angiotensin-(1–7) inhibits epidermal growth factor receptor transactivation via a Mas receptor-dependent pathway. Br J Pharmacol. 2012b;165: 1390–1400. 21806601 10.1111/j.1476-5381.2011.01613.x PMC3372724

[36] Akhtar S, Chandrasekhar B, Attur S, Yousif MH, Benter IF. On the nanotoxicity of PAMAM dendrimers: Superfect stimulates the EGFR-ERK1/2 signal transduction pathway via an oxidative stress-dependent mechanism in HEK 293 cells. Int J Pharm. 2013b;448(1): 239–246. 23538097 10.1016/j.ijpharm.2013.03.039

[37] Omidi Y, Hollins AJ, Benboubetra M, Drayton R, Benter IF, Akhtar S. Toxicogenomics of non-viral vectors for gene therapy: a microarray study of lipofectin- and oligofectamine-induced gene expression changes in human epithelial cells. J. Drug Target. 2003;11: 311–323. 14668052 10.1080/10611860310001636908

[38] Chauhan AS, Diwan PV, Jain NK, Tomalia DA. Unexpected in vivo anti-inflammatory activity observed for simple, surface functionalized poly(amidoamine) dendrimers. Biomacromolecules. 2009;10: 1195–1202. 10.1021/bm9000298 19348417

[39] Li Y, Zeng X, Wang S, Sun Y, Wang Z, Fan J, Song P, et al. Inhibition of autophagy protects against PAMAM dendrimers-induced hepatotoxicity. Nanotoxicology. 2014;1–12. [Epub ahead of print] . 24983897 10.3109/17435390.2014.930533

[40] Benter IF, Canatan H, Benboubetra M, Yousif MH, Akhtar S. Global upregulation of gene expression associated with renal dysfunction in DOCA-salt-induced hypertensive rats occurs via signaling cascades involving epidermal growth factor receptor: a microarray analysis. Vascul Pharmacol. 2009;51(2–3): 101–109. 10.1016/j.vph.2009.04.004 19410658

[41] Zhang MZ, Wang Y, Paueksakon P, Harris RC. Epidermal growth factor receptor inhibition slows progression of diabetic nephropathy in association with a decrease in endoplasmic reticulum stress and an increase in autophagy. Diabetes. 2014;63(6): 2063–2072. 10.2337/db13-1279 24705402 PMC4030104

[42] Advani A, Wiggins KJ, Cox AJ, Zhang Y, Gilbert RE, Kelly DJ. Inhibition of the epidermal growth factor receptor preserves podocytes and attenuates albuminuria in experimental diabetic nephropathy. Nephrology (Carlton). 2011;16(6): 573–581. 21342330 10.1111/j.1440-1797.2011.01451.x

[43] Shcharbin D, Janaszewska A, Klajnert-Maculewicz B, Ziemba B, Dzmitruk V, Halets I. How to study dendrimers and dendriplexes III. Biodistribution, pharmacokinetics and toxicity in vivo. J Control Release. 2014;181: 40–52. 10.1016/j.jconrel.2014.02.021 24607663

[44] Sadekar S, Ray A, Janàt-Amsbury M, Peterson CM, Ghandehari H. Comparative biodistribution of PAMAM dendrimers and HPMA copolymers in ovarian-tumor-bearing mice. Biomacromolecules. 2011;12(1): 88–96. Erratum in: Biomacromolecules. 2011;12(9): 3351. 10.1021/bm101046d 21128624 PMC3476841

[45] Alaoui-Jamali MA, Morand GB, da Silva SD. ErbB polymorphisms: insights and implications for response to targeted cancer therapeutics. Front Genet. 2015;4(6): 17. 10.3389/fgene.2015.00017 PMC4316710 25699077

[46] Price KA, Cohen EE. Mechanisms of and therapeutic approaches for overcoming resistance to epidermal growth factor receptor (EGFR)-targeted therapy in squamous cell carcinoma of the head and neck (SCCHN). Oral Oncol. 2015;51(5): 399–408. 10.1016/j.oraloncology.2015.01.018 25725588

[47] Han J, Shuvaev VV, Muzykantov VR. Targeted interception of signaling reactive oxygen species in the vascular endothelium. Ther. Deliv. 2012;3: 263–276. 22834201 10.4155/tde.11.151 PMC5333711

[48] Wilcox CS. Effects of tempol and redox-cycling nitroxides in models of oxidative stress. Pharmacol Ther. 2010;126: 119–145. 10.1016/j.pharmthera.2010.01.003 20153367 PMC2854323

[49] Sur S, Agrawal DK. Transactivation of EGFR by G protein-coupled receptor in the pathophysiology of intimal hyperplasia. Curr Vasc Pharmacol. 2014;12(2):190–201. 24568153 10.2174/1570161112666140226123745

[50] Kovacs E, Zorn JA, Huang Y, Barros T, Kuriyan J. A Structural Perspective on the Regulation of the Epidermal Growth Factor Receptor. Annu Rev Biochem. 2015 [Epub ahead of print] . 25621509 10.1146/annurev-biochem-060614-034402 PMC4452390

[51] Arkhipov A, Shan Y, Das R, Endres NF, Eastwood MP, Wemmer DE, et al Architecture and membrane interactions of the EGF receptor. Cell. 2013;152(3): 557–569. 10.1016/j.cell.2012.12.030 23374350 PMC3680629

[52] Lemmon MA, Schlessinger J, Ferguson KM. The EGFR family: not so prototypical receptor tyrosine kinases. Cold Spring Harb Perspect Biol. 2014;6(4): a020768 10.1101/cshperspect.a020768 24691965 PMC3970421

[53] Avaritt BR, Swaan PW. Intracellular Ca2+ release mediates cationic but not anionic poly(amidoamine) (PAMAM) dendrimer-induced tight junction modulation. Pharm Res. 2014;31(9): 2429–2438. 10.1007/s11095-014-1338-y 24648136

[54] Lill NL, Sever NI. Where EGF receptors transmit their signals. Sci Signal. 2012;5: pe41 23012653 10.1126/scisignal.2003341 PMC3507515

[55] Omerovic J, Hammond D E, Prior IA. Clague MJ. Global Snapshot of the Influence of Endocytosis upon EGF Receptor Signaling Output. J. Proteome. Res. 2012;11: 5157–5166. 10.1021/pr3007304 22974187

[56] Albertazzi L, Serresi M, Albanese A, Beltram F. Dendrimer internalization and intracellular trafficking in living cells. Mol. Pharm. 2010;7: 680–688. 10.1021/mp9002464 20394437

[57] Seib FP, Jones AT, Duncan R. Comparison of the endocytic properties of linear and branched PEIs, and cationic PAMAM dendrimers in B16f10 melanoma cells. J Control Release. 2007;117: 291–300. 17210200 10.1016/j.jconrel.2006.10.020

[58] Cui J, Hu YF, Feng XM, Tian T, Guo YH, Ma JW, et al EGFR inhibitors and autophagy in cancer treatment. Tumour Biol. 2014;35(12): 11701–11709. 10.1007/s13277-014-2660-z 25293518

[59] Lee JH, Cha KE, Kim MS, Hong HW, Chung DJ, Ryu G, et al Nanosized polyamidoamine (PAMAM) dendrimer-induced apoptosis mediated by mitochondrial dysfunction. Toxicol. Lett. 2009;190: 202–207. 10.1016/j.toxlet.2009.07.018 19643170

[60] Mukherjee SP, Davoren M, Byrne HJ. In vitro mammalian cytotoxicological study of PAMAM dendrimers—towards quantitative structure activity relationships. Toxicol. In Vitro. 2010a; 24: 169–177. 19778601 10.1016/j.tiv.2009.09.014

[61] Mukherjee SP, Lyng FM, Garcia A, Davoren M, Byrne HJ. Mechanistic studies of in vitro cytotoxicity of poly(amidoamine) dendrimers in mammalian cells. Toxicol. Appl. Pharmacol. 2010b;248: 259–268. 20736030 10.1016/j.taap.2010.08.016

[62] Naha PC, Davoren M, Lyng FM, Byrne HJ. Reactive oxygen species (ROS) induced cytokine production and cytotoxicity of PAMAM dendrimers in J774A.1 cells. Toxicol. Appl. Pharmacol. 2010;246: 91–99. 10.1016/j.taap.2010.04.014 20420846

[63] Kanwar YS, Wada J, Sun L, Xie P, Wallner EI, Chen S, et al Diabetic nephropathy: mechanisms of renal disease progression. Exp Biol Med (Maywood). 2008;233(1): 4–11. 18156300 10.3181/0705-MR-134

[64] François H, Placier S, Flamant M, Tharaux PL, Chansel D, Dussaule JC, et al Prevention of renal vascular and glomerular fibrosis by epidermal growth factor receptor inhibition. FASEB J. 2004;18(7): 926–928. 15033924 10.1096/fj.03-0702fje

[65] Kabanov AV. Polymer genomics: an insight into pharmacology and toxicology of nanomedicines. Adv. Drug Deliv. 2006;58: 1597–1621. 10.1016/j.addr.2006.09.019 PMC1853357 17126450

[66] Kuo JH, Liou MJ, Chiu HC. Evaluating the gene-expression profiles of HeLa cancer cells treated with activated and nonactivated poly(amidoamine) dendrimers, and their DNA complexes. Mol Pharm. 2010;7: 805–814. 10.1021/mp900303s 20394435

[67] Jones CF, Campbell RA, Franks Z, Gibson CC, Thiagarajan G, Vieira-de-Abreu A, et al Cationic PAMAM dendrimers disrupt key platelet functions. Mol. Pharm. 2012;9(6): 1599–1611. 10.1021/mp2006054 22497592 PMC3367133

[68] Sun Y, Guo F, Zou Z, Li C, Hong X, Zhao Y, et al Cationic nanoparticles directly bind angiotensin-converting enzyme 2 and induce acute lung injury in mice. Part Fibre Toxicol. 2015;12(1): 4. 25890286 10.1186/s12989-015-0080-x PMC4395934

[69] Benter IF, Yousif MH, Griffiths SM, Benboubetra M, Akhtar S. Epidermal growth factor receptor tyrosine kinase-mediated signalling contributes to diabetes-induced vascular dysfunction in the mesenteric bed. Br J Pharmacol. 2005;145: 829–836. 15852031 10.1038/sj.bjp.0706238 PMC1576192

